# Downregulation of IGFBP5 contributes to replicative senescence via ERK2 activation in mouse embryonic fibroblasts

**DOI:** 10.18632/aging.203999

**Published:** 2022-04-04

**Authors:** Iyori Nojima, Ryusuke Hosoda, Yuki Toda, Yoshiki Saito, Naohiro Ueda, Kouhei Horimoto, Naotoshi Iwahara, Yoshiyuki Horio, Atsushi Kuno

**Affiliations:** 1 Department of Pharmacology, Sapporo Medical University School of Medicine, Sapporo, Japan

**Keywords:** IGFBP5, replicative senescence, mouse embryonic fibroblasts, ERK2, ERK1

## Abstract

Insulin-like growth factor (IGF)-binding proteins (IGFBPs) are secretory proteins that regulate IGF signaling. In this study, we investigated the role of IGFBP5 in replicative senescence in embryonic mouse fibroblasts (MEFs). During passages according to the 3T3 method, MEFs underwent senescence after the 5th passage (P5) based on cell growth arrest, an increase in the number of cells positive for senescence-associated β-galactosidase (SA-β-GAL) staining, and upregulation of p16 and p19. In P8 MEFs, IGFBP5 mRNA level was markedly reduced compared with that in P2 MEFs. Downregulation of IGFBP5 via siRNA in P2 MEFs increased the number of SA-β-GAL-positive cells, upregulated p16 and p19, and inhibited cell growth. Incubation of MEFs with IGFBP5 during serial passage increased the cumulative population doubling and decreased SA-β-GAL positivity compared with those in vehicle-treated cells. IGFBP5 knockdown in P2 MEFs increased phosphorylation levels of ERK1 and ERK2. Silencing of ERK2, but not that of ERK1, blocked the increase in the number of SA-β-GAL-positive cells in IGFBP5-knockdown cells. The reduction in the cell number and upregulation of p16 and p21 in IGFBP5-knockdown cells were attenuated by ERK2 knockdown. Our results suggest that downregulation of IGFBP5 during serial passage contributes to replicative senescence via ERK2 in MEFs.

## INTRODUCTION

Cellular senescence is defined as a permanent state of growth arrest that is associated with enlarged and flattened morphologies, upregulation of cyclin-dependent kinase (CDK) inhibitors including p16, p19, and p21, and accumulation of lysosomes and nuclear expansion [1]. Physiological roles of cellular senescence include normal development [2], wound healing [3], and anti-tumor effects [4]. On the other hand, senescent cells accumulate in an organ during organismal aging. A recent study showed that removal of p16-positive senescent cells attenuated aging-associated tissue damage and improved organismal healthspan [5], indicating that senescent cells contribute to various aging-associated pathologies [1]. Therefore, an understanding of the molecular basis of cellular senescence will reveal how aging-related diseases develop and progress. There are currently several experimental models of cellular senescence. Hayflick and Moorhead observed that primary human fibroblasts in culture exhibit a limited proliferative capacity [6]. This growth arrest during passages is called replicative senescence. Cellular senescence is also induced by activation of oncogenic signaling such as Ras, ultraviolet, γ-irradiation, hydrogen peroxide, and chemotherapy agents. Replicative senescence in mouse embryonic fibroblasts (MEFs) is a widely used model for research on cellular senescence. The advantages of the replicative senescence model in MEFs include easy isolation of cells from mouse embryos and more rapid termination of cell proliferation than that of human fibroblasts.

Insulin/insulin-like growth factor-1 (IGF-1) signaling has been reported to play key roles in organismal lifespan [7, 8] as well as cellular senescence [9, 10]. Suppression of insulin/IGF-1 signaling extends lifespan. In *C. elegans*, insulin/IGF1 signaling negatively regulates lifespan via activating Akt, which phosphorylates and inactivates DAF-16, a homologue of mammalian FOXO transcription factors [8, 11]. In mammals, suppression of FOXO3a, the mammalian DAF-16 homologue, by activated Akt leads to repression of anti-oxidative enzymes, which causes enhanced oxidative stress and cellular senescence [9, 10]. The activity of Akt was increased in senescent human endothelial cells [9] and human dermal fibroblasts [12]. Inhibition of Akt activity delayed the entry to senescence in human endothelial cells [9] and MEFs [10]. In contrast, constitutive activation of Akt accelerated replicative senescence via a p53/p21-dependent pathway [9]. Insulin/IGF-1 signaling also activates mitogen-activated protein kinases including extracellular signal-regulated kinases 1 and 2 (ERK1 and ERK2) via the Ras/Raf/MEK signaling cascade. A high level of Raf activity induces cell cycle arrest via p16 and p21 expression [13, 14]. Phosphorylation and activation of ERK1 and ERK2 are involved in oncogenic Ras-induced senescence as well as replicative senescence [15]. ERK2 contributes to the upregulation of p16 in Ras-induced senescence via the Ets transcription factor in MEFs [16]. ERK has also been reported to induce p21 transcription via the Ets family transcription factor ELK1 [17]. Inhibition of Ras/ERK/Ets signaling leads to increased lifespan in Drosophila [18]. Therefore, Akt and ERK pathways are important determinants of cellular senescence as well as organismal aging downstream of the IGF-1 receptor.

Insulin/IGF-1 signaling is regulated by a variety of components. IGF-binding proteins (IGFBPs) are secretory proteins that bind to IGF-1 and IGF-2 in the circulation [19]. The IGFBP family generally consists of six members of IGFBPs (IGFBP1~6). Another member, IGFBP7, has weak binding affinity to IGFs [20]. Binding of IGFBPs to IGFs extends the circulating half-life time of IGFs, leading to enhancement of IGF signaling. On the other hand, binding of IGFBPs to IGFs blocks the ability of IGFs to activate IGF receptors, resulting in suppression of IGF signaling [19]. IGFBPs also localize intracellularly and regulate cellular function in an IGF-independent manner [19, 21, 22]. For example, nuclear IGFBP5 may regulate gene expression via its transactivation activity of the N-terminal region [22].

Among the IGFBP family members, IGFBP5 is the most highly conserved across species and regulates a variety of cellular processes including cell survival, proliferation, and differentiation [23]. IGFBP5 is expressed in a variety of tissues including the lung, bone, muscle, testis, ovary, and kidney [24]. There have been some studies showing age- or disease-associated downregulation of IGFBP5 levels in humans. IGFBP5 levels in the serum and bone [25] and in skeletal muscle [26] were found to be lower in aged people than in young adults. It has been reported that serum levels of IGFBP5 are decreased in patients with type 1 and type 2 diabetes [27] and in patients with hip fractures [28]. In addition, IGFBP5 level has been shown to be positively correlated with bone mineral density in the femoral neck [27, 28]. Single knockout of IGFBP5 in mice had a minimal effect on muscle size and bone formation due to compensation by the other IGFBPs [29]. However, triple knockout of IGFBP3, IGFBP4, and IGFBP5 resulted in reduced muscle mass [29]. This indicates that IGFBPs play key roles in the maintenance of muscle mass and suggests that the age- or disease-related decline in IGFBP5 level may be involved in age-related pathologies.

Previous studies have shown that cellular senescence is promoted by IGFBP2 [30], IGFBP3 [31], IGFBP4 [32], IGFBP5 [33], and IGFBP7 [32] and is suppressed by IGFBP1 [34] and IGFBP6 [35]. In contrast to aging-related downregulation in humans as described above, IGFBP5 has been reported to be upregulated during passages and to contribute to replicative senescence via a p53-dependent pathway in human umbilical endothelial cells (HUVECs) [33]. Upregulated IGFBP5 induced by IL-6/IL-6 receptor mediates premature senescence in human fibroblasts [36]. Therefore, upregulation of IGFBP5 induced by senescence-inducing stimuli contributes to cellular senescence in human cell models. However, there has been no study in which the roles of IGFBPs in cellular senescence in MEFs were evaluated.

In the present study, we focused on IGFBPs in the replicative senescence model of MEFs. We found that the senescence-associated change in IGFBP5 expression was the greatest among IGFBP family members and that its expression was downregulated with serial passage. We showed that downregulation of IGFBP5 expression by knockdown induced premature senescence in young MEFs that was associated with increased levels of phosphorylated ERK1 (pERK1) and pERK2. Silencing of ERK2 blocked the senescence phenotypes induced by IGFBP5 knockdown. These findings suggest that downregulation of IGFBP5 contributes to replicative senescence via ERK2 activation in MEFs.

## RESULTS

### MEFs undergo replicative senescence during serial passage

**Figure 1:**
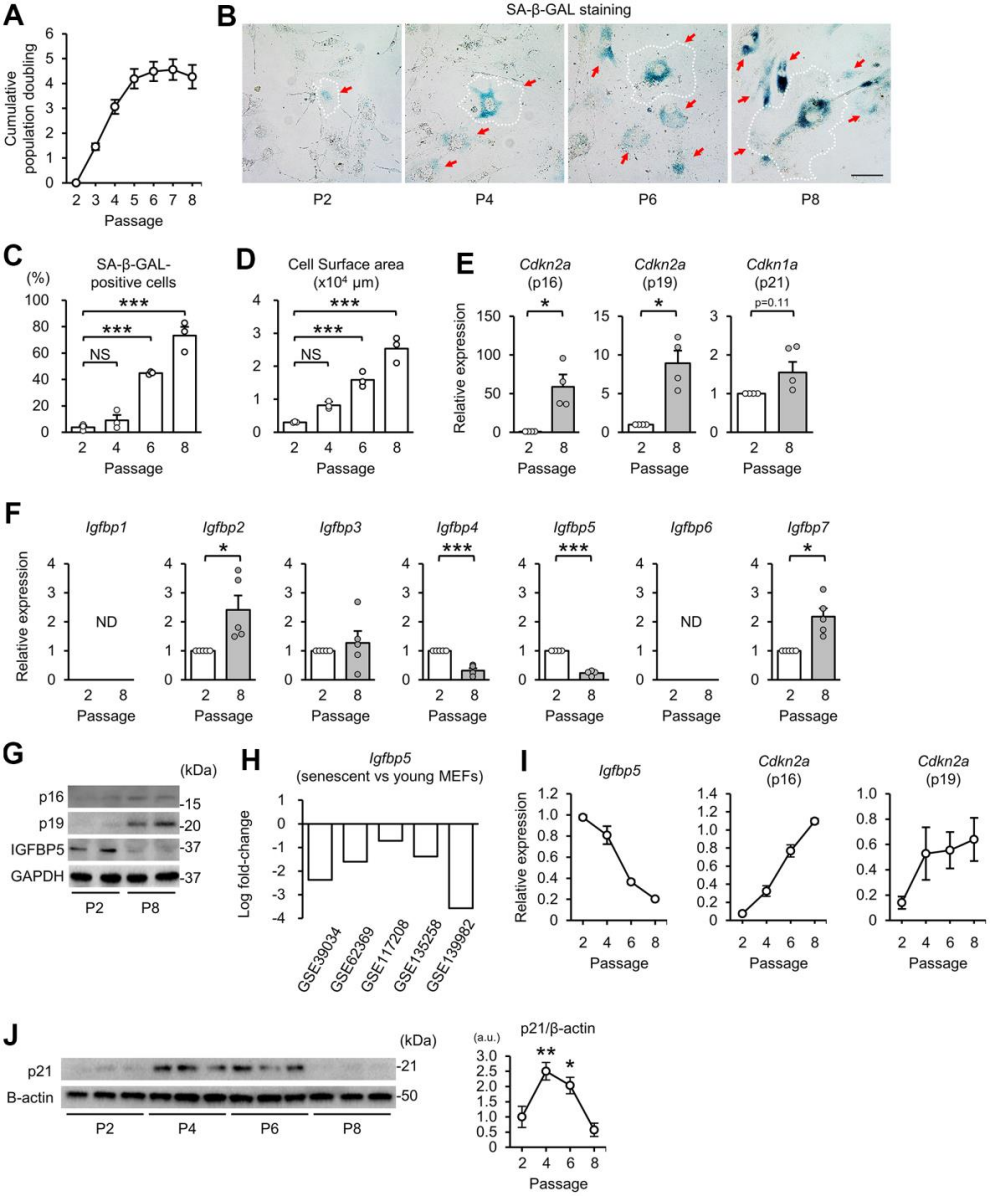
**Downregulation of IGFBP5 in senescent MEFs.** (**A**) Cumulative population doubling of MEFs during the 3T3 passage. N=8. (**B**) Representative images of senescence-associated β-galactosidase (SA-β-GAL) staining in MEFs at the 2nd (P2), 4th (P4), 6th (P6), and 8th (P8) passages. A white dotted line in each field was added to visualize the representative outline of the cell at each passage. Red arrows indicate cells positive for SA-β-GAL staining. Scale bar, 100 μm. (**C**) Summary data of the percentage of SA-β-GAL-positive cells. N=3 in each group. ***P<0.001 by one-way repeated measures ANOVA with a Student-Newman-Keuls test for multiple comparisons. (**D**) Summary data of cell surface areas. N=3. ***P<0.001 by one-way repeated measures ANOVA with a Student-Newman-Keuls test for multiple comparisons. (**E**) Levels of *Cdkn2a* (p16 and p19) and *Cdkn1a* (p21) mRNA in MEFs at P2 and P8. N=4 in each group. *P<0.05 by paired Student’s t-test. (**F**) Gene expression of IGFBPs in P2 and P8 MEFs. ND: not detected. N=5 in each group. *P<0.05, ***P<0.001 by paired Student’s t-test. (**G**) Representative immunoblots for p16, p19 and IGFBP5 in P2 and P8 MEFs. kDa: kilodalton. (**H**) Changes in *Igfbp5* expression of senescent MEFs compared with young MEFs in five datasets from the Gene Expression Omnibus. (**I**) Changes in *Igfbp5* and *Cdkn2a* (p16 and p19) mRNA levels during serial passage. N=4 in each passage. (**J**) Representative immunoblots (left) and quantitative data (right) for p21 and β-actin in P2, P4, P6, and P8 MEFs. N=3 in each passage from three independent experiments. *P<0.05, **P<0.01 by one-way repeated measures ANOVA with a Student-Newman-Keuls test. a.u.: arbitrary unit. Data are represented as mean +/- SEM. NS: not significant.

First, we prepared a model of replicative senescence in MEFs. Cumulative population doubling during the 3T3 passage in MEFs [37] increased from passage 2 (P2) to P5 and then reached a plateau after P5 (Figure 1A). Cells positive for senescence-associated β-galactosidase (SA-β-GAL) staining were increased in MEFs at P6 and P8 compared with that at P2 (Figure 1B, 1C). An enlarge and flattened cell shape is a hallmark of senescent cells [1]. Cell surface areas started to enlarge at P4 and were further increased in P6 and P8 cells (Figure 1B, 1D). Transcript levels of the CDK inhibitors p16 and p19, which are transcribed from *Cdkn2a*, were upregulated at P8 compared with those at P2 (Figure 1E). Expression of *Cdkn1a* (p21) tended to be increased in P8 MEFs. These findings indicate induction of replicative senescence after P5.

### IGFBP5 expression is downregulated during serial passage

We next compared the expression levels of IGFBPs in P2 and P8 MEFs. Reverse transcription-quantitative polymerase chain reaction (RT-qPCR) analyses demonstrated that *Igfbp2* and *Igfbp7* mRNA levels were upregulated and that *Igfbp4* and *Igfbp5* mRNA levels were downregulated in P8 MEFs compared with those in P2 MEFs (Figure 1F). *Igfbp1* and *Igfbp6* transcripts were not detected in either P2 or P8 MEFs. In senescent P8 MEFs, the level of *Igfbp5* expression was reduced to 23% of that in P2 MEFs, which was the largest change among the IGFBPs. The protein level of IGFBP5 in cellular lysates was also decreased in P8 MEFs compared with that in P2 MEFs. Protein levels of p16 and p19 were increased in P8 MEFs, as we expected (Figure 1G). In addition, five datasets of DNA microarray and RNA sequencing from the Gene Expression Omnibus (GEO, http://www.ncbi.nlm.nih.gov/geo/) [38–42] demonstrated that IGFBP5 expression was commonly downregulated in senescent MEFs (Figure 1H). Therefore, we focused on the role of IGFBP5 in cellular senescence in MEFs. The time course of the change in *Igfbp5* mRNA levels during serial passage revealed that downregulation started before proliferation arrest (Figure 1I). There was a correlation between downregulation of *Igfbp5* and upregulation of *Cdkn2a* (p16 and p19) during the 3T3 passage (Figure 1I). Protein levels of p21 were upregulated at P4 and P6 and then returned to the level of P2 MEFs at P8 (Figure 1J).

### Knockdown of IGFBP5 induces premature senescence in P2 MEFs

**Figure 2:**
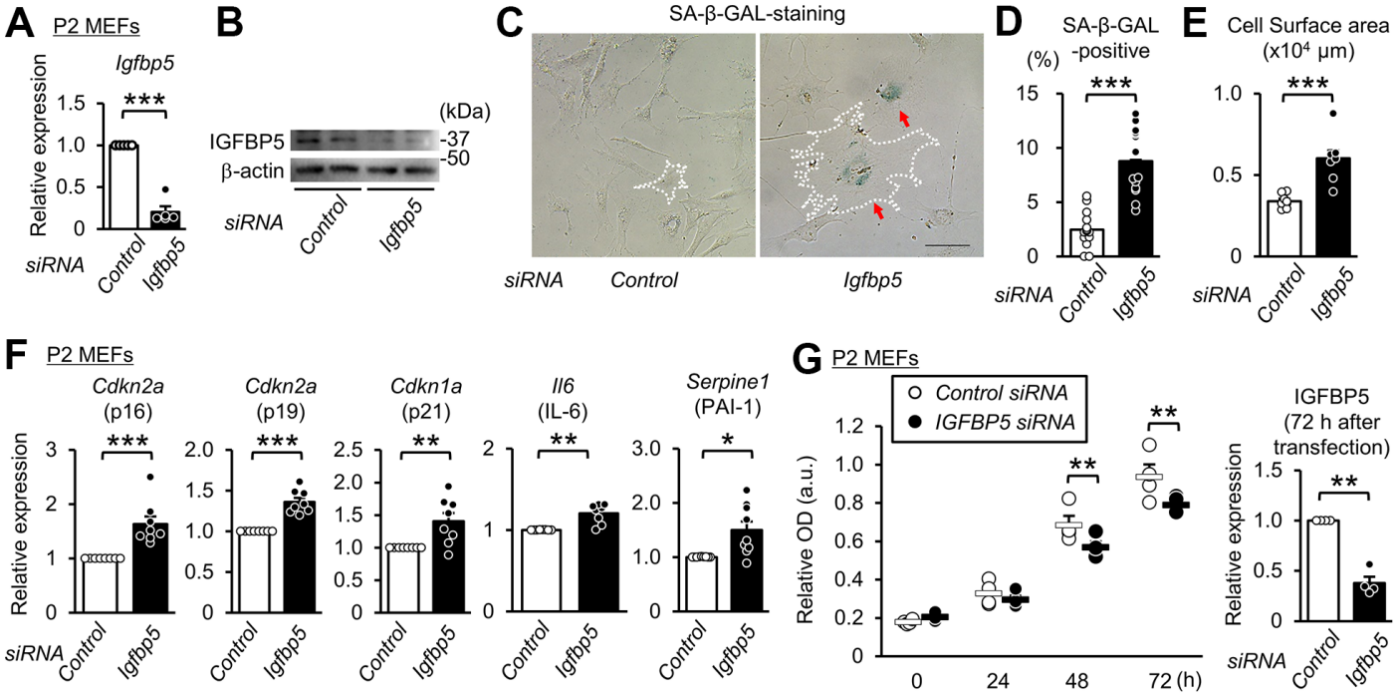
**Knockdown of IGFBP5 induces premature senescence in young MEFs.** (**A**) Levels of *Igfbp5* mRNA in P2 MEFs 48 h after transfection with control siRNA and siRNA against *Igfbp5*. N=5 in each treatment. ***P<0.001 by paired Student’s t-test. (**B**) Representative immunoblot for IGFBP5 in P2 MEFs transfected with control siRNA and siRNA against *Igfbp5*. kDa: kilodalton. (**C**) Representative images of SA-β-GAL staining in cells transfected with control siRNA or *Igfbp5* siRNA. A white dotted line in each field was added to visualize the representative outline of the cell. Red arrows indicate cells positive for SA-β-GAL staining. Scale bar, 100 μm. (**D**) Summary data of the percentage of SA-β-GAL-positive cells. N=14 from two independent experiments in each treatment. ***P<0.001 by unpaired Student’s t-test. (**E**) Summary data of cell surface areas. N=8 from two independent experiments. ***P<0.001 by unpaired Student’s t-test. (**F**) Levels of *Cdkn2a* (p16 and p19), *Cdkn1a* (p21), *Il6* and *Serpine1* (PAI-1) mRNA in P2 MEFs transfected with control siRNA or siRNA against *Igfbp5*. N=8-9 in each treatment. *P<0.05, **P<0.01, ***P<0.001 by paired Student’s t-test. (**G**) (Left) Cell proliferation in P2 MEFs transfected with control siRNA and siRNA against *Igfbp5* determined by Cell Counting Kit-8. N=4 in each treatment. **P<0.01 by two-way repeated measures ANOVA with a Student-Newman-Keuls test. (Right) Level of *Igfbp5* mRNA 72 h after transfection. **P<0.01 by paired Student’s t-test. Data are represented as mean +/- SEM.

To determine whether downregulation of IGFBP5 contributes to cellular senescence, we analyzed the effects of knockdown of *Igfbp5* on indices of cellular senescence in P2 MEFs. Transfection of small interfering RNA (siRNA) against *Igfbp5* decreased the *Igfbp5* mRNA level to 20% of that in cells transfected with control siRNA (Figure 2A). The protein level of IGFBP5 was markedly reduced by siRNA against *Igfbp5* (Figure 2B). The percentage of SA-β-GAL-positive cells was approximately 3.5-fold higher in cells transfected with *Igfbp5* siRNA than in cells transfected with control siRNA (Figure 2C, 2D). Knockdown of IGFBP5 by two siRNAs with different sequences also increased the percentage of SA-β-GAL-positive cells (Supplementary Figure 1). Knockdown of IGFBP5 also increased cell surface areas (Figure 2C, 2E). Levels of *Cdkn2a* (p16 and p19) and *Cdkn1a* (p21) mRNA were increased by IGFBP5 knockdown (Figure 2F). Senescent cells secrete a variety of bioactive proteins such as cytokines, chemokines, and proteases, which affect surrounding cells [43]. Levels of *Il6* (interleukin-6) and *Serpine1* (plasminogen activator inhibitor Type 1) mRNA were increased in IGFBP5-knockdown cells (Figure 2F). The number of cells was significantly decreased at 48 and 72 h after transfection with *Igfbp5* siRNA compared to that after transfection with control siRNA (Figure 2G). These findings indicate that downregulation of IGFBP5 induced cellular senescence in P2 MEFs.

IGFBP5 is a secretary protein and is known to work also inside the cell. However, it is unknown whether intracellular or extracellular IGFBP5 contributes to suppression of cellular senescence. To determine whether intrinsic IGFBP5 is related intracellularly to suppression of the senescence phenotype, we visualized cells transfected with siRNA targeting *Igfbp5* by labeling siRNA with fluorescence into P2 MEFs and performed SA-β-GAL staining. Fluorescence microscopy showed that 42% of the cells were positive for FAM after transfection. There was no significant difference in the percentage of SA-β-GAL-positive cells between FAM-negative MEFs and FAM-positive MEFs (Supplementary Figure 2). This finding suggests that the reduction in intrinsic IGFBP5 level inside the cell does not induce senescence.

### Supplementation of IGFBP5 delays the entry to replicative senescence

**Figure 3:**
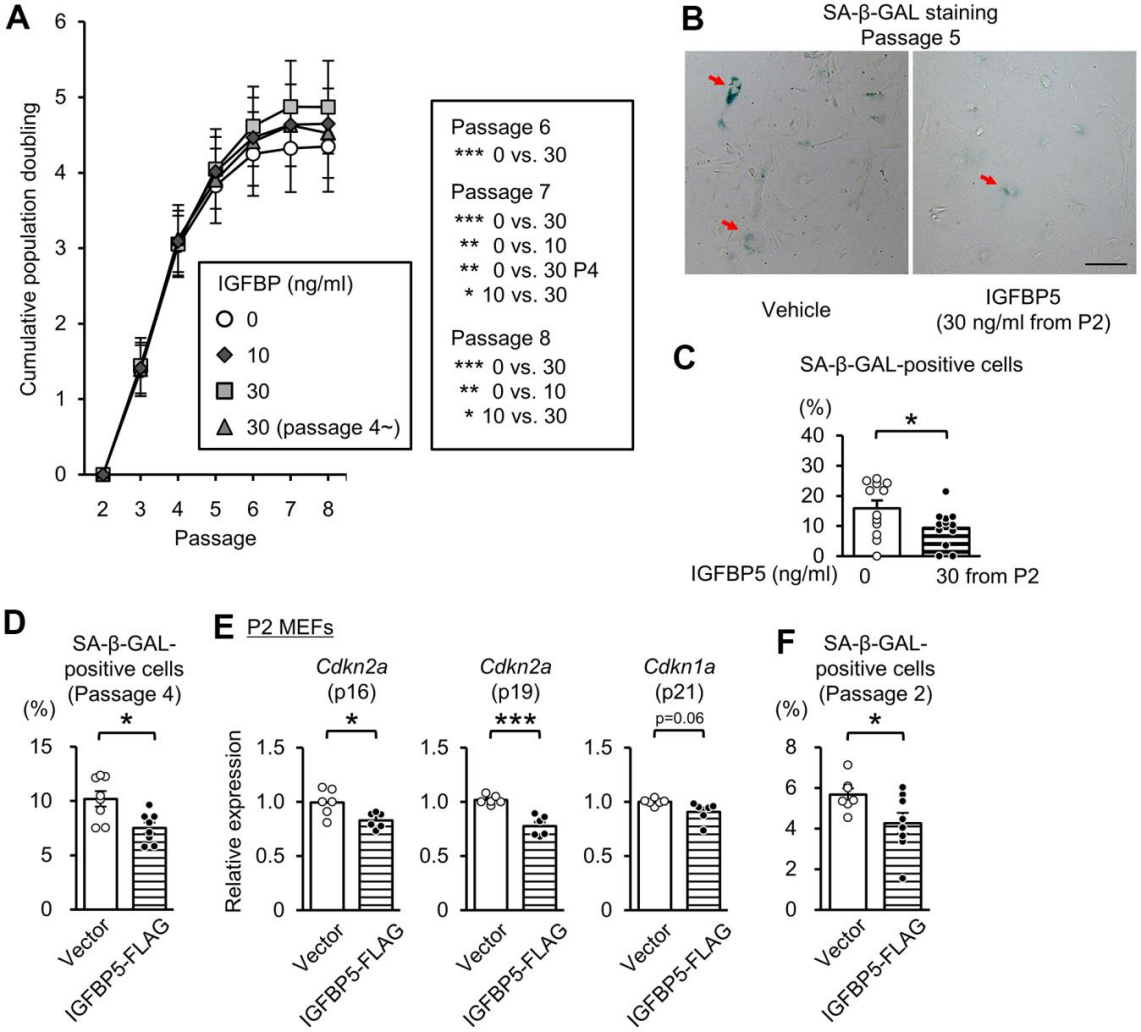
**Effects of exogenous IGFBP5 on replicative senescence in MEFs.** (**A**) Cumulative population doubling in MEFs passaged with a 3T3 method that were treated with a vehicle or IGFBP5 (10, 30 ng/ml) starting at P2 or 30 ng/ml IGFBP5 starting P4. N=5 in each treatment. *P<0.05, **P<0.01, ***P<0.001 by two-way repeated measures ANOVA with a Student-Newman-Keuls test. (**B**) Representative images of SA-β-GAL staining in MEFs at the 5th passage treated with the vehicle or IGFBP5 (30 ng/ml) starting at P2. Red arrows indicate cells positive for SA-β-GAL staining. Scale bar, 100 μm. (**C**) Summary data of the percentage of SA-β-GAL-positive cells. N=12 from three independent experiments in each treatment. *P<0.05 by unpaired Student’s t-test. (**D**) Summary data of the percentage of SA-β-GAL-positive cells in P4 MEFs transfected with an empty vector or IGFBP5-FLAG. N=8 from two independent experiments in each treatment. *P<0.05 by unpaired Student’s t-test. (**E**) Levels of *Cdkn2a* (p16 and p19) and *Cdkn1a* (p21) mRNA in P2 MEFs transfected with an empty vector or IGFBP5-FLAG. N=6 in each treatment. *P<0.05, ***P<0.001 by unpaired Student’s t-test. (**F**) Summary data of the percentage of SA-β-GAL-positive cells in P2 MEFs transfected with an empty vector or IGFBP5-FLAG. N=7-8 from two independent experiments in each treatment. *P<0.05 by unpaired Student’s t-test. Data are represented as mean +/- SEM.

To determine the role of extracellular IGFBP5, we next investigated the effects of exogenous supplementation of IGFBP5 on replicative senescence. IGFBP5-FLAG was purified from a medium of COS7 cells expressing IGFBP5-FLAG. The medium from COS7 cells expressing IGFBP5-FLAG inhibited IGF-1-induced Akt phosphorylation (Supplementary Figure 3). We incubated MEFs with a vehicle and with 10 ng/ml and 30 ng/ml IGFBP5 starting from P2. MEFs were also incubated with 30 ng/ml IGFBP5 starting from P4. Incubation with IGFBP5 at 30 ng/ml from P2 increased the cumulative population doubling levels at P6, P7, and P8 compared with those in vehicle-treated control cells (Figure 3A). Incubation with 10 ng/ml IGFBP5 from P2 and 30 ng/ml IGFBP5 from P4 also increased the cumulative population doubling, but the effects were less than that of 30 ng/ml IGFBP5 from P2 (Figure 3A). The percentage of cells positive for SA-β-GAL at P5 was decreased by treatment with 30 ng/ml IGFBP5 from P2 (Figure 3B, 3C). These findings suggest that extracellularly supplemented IGFBP5 plays a role in suppression of replicative senescence. Levels of *Cdkn2a* (p16 and p19) in P6 MEFs tended to be reduced by incubation with 30 ng/ml IGFBP5 from P2, but the difference did not reach statistical significance. *Cdkn1a* (p21) mRNA was not decreased by exogenous IGFBP5 at P6 (Supplementary Figure 4). The expression levels were not changed by incubation with either IGFBP5 at 10 ng/ml from P2 or 30 ng/ml from P4.

We further evaluated whether overexpression of IGFBP5 in MEFs reduces the senescence phenotype. Transfection of an expression vector of IGFBP5-FLAG into P4 MEFs reduced the percentage of cells positive for SA-β-GAL staining (Figure 3D). Overexpression of IGFBP5 in P2 MEFs significantly reduced levels of *Cdkn2a* (p16 and p19) and tended to reduce *Cdkn1a* (p21) mRNA level (Supplementary Figure 4A and Figure 3E). The percentage of SA-β-GAL-positive cells was also reduced by overexpression of IGFBP5 in P2 MEFs (Figure 3F).

### Knockdown of IGFBP5 did not affect expression levels of p16 repressors

We evaluated whether knockdown of IGFBP5 affects regulators of p16 expression. Expression of p16 is inhibited by polycomb repressor complexes 1/2 (PRC1/2). Ezh2, a component of PRC2, is a methyltransferase specific to a histone H3 lysine 27 (H3K27) and epigenetically represses the p16 gene via H3K27 trimethylation at the p16 locus [44]. Expression of Ezh2 has been reported to be downregulated in senescent MEFs, which leads to p16 expression [44]. In the present study, *Ezh2* mRNA level was reduced in P8 MEFs compared with that in P2 MEFs (Supplementary Figure 5A). On the other hand, the level was not changed by IGFBP5 knockdown. Messenger RNA levels of *Bmi1* [45] and *Id1* [46], other known p16 repressors, were unchanged in P8 MEFs and IGFBP5-knockdown cells (Supplementary Figure 5A, 5B). Therefore, knockdown of IGFBP5 did not affect expression levels of these negative regulators of p16 expression.

### Knockdown of IGFBP5 increased phosphorylation of ERK1/2 but not that of Akt

**Figure 4:**
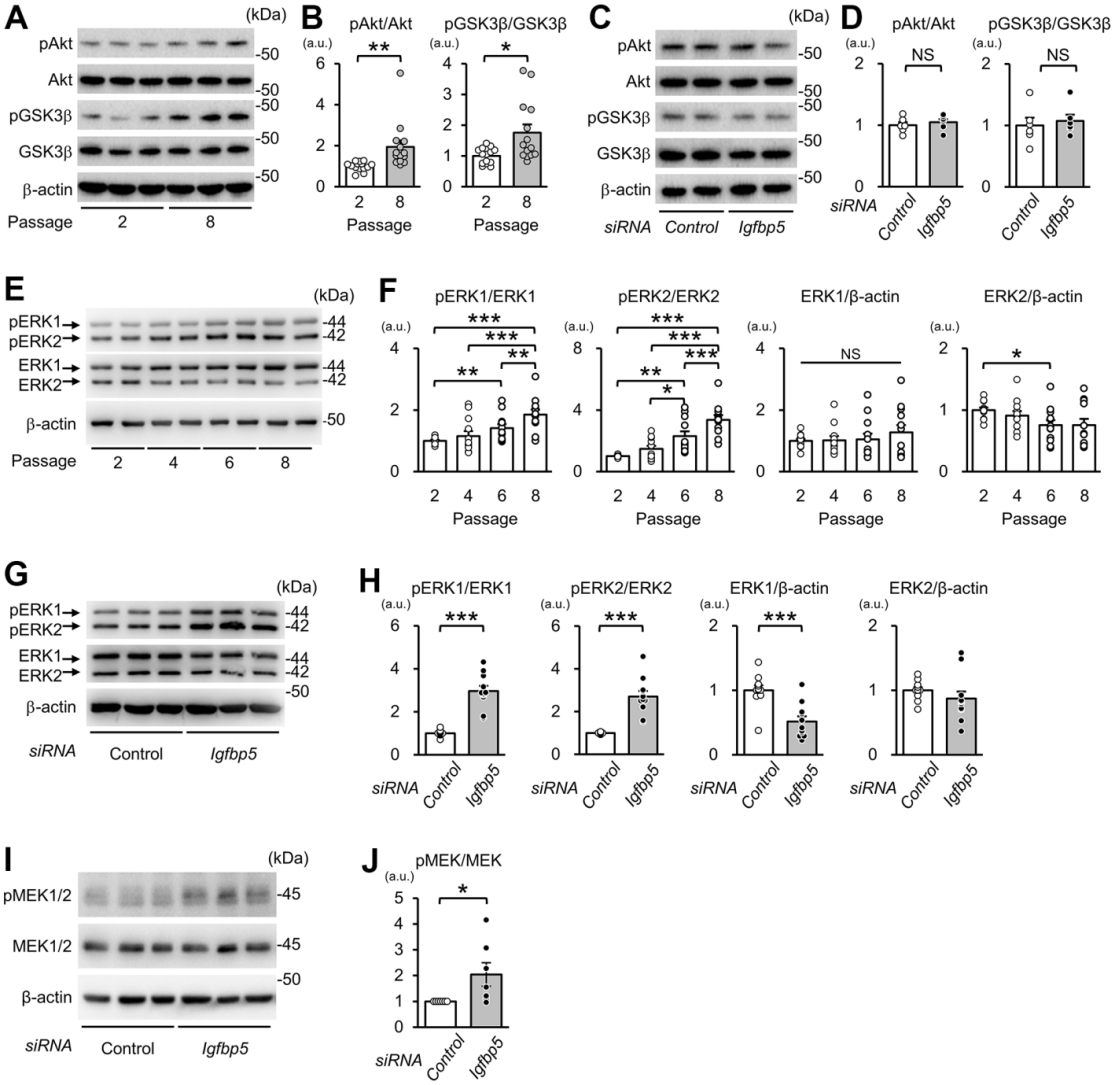
**Effects of serial passage and IGFBP5 knockdown on Akt and ERK phosphorylation in MEFs.** (**A**) Representative immunoblots for phospho-Ser473-Akt (pAkt), Akt, phospho-Ser9-GSK3β (pGSK3β), GSK3β, and β-actin in P2 and P8 MEFs. (**B**) Quantitative data for pAkt and pGSK3β levels normalized to level of the corresponding total protein. N=14 from four independent experiments. *P<0.05, **P<0.01 by unpaired Student’s t-test. (**C**) Representative immunoblots for pAkt, Akt, pGSK3β and GSK3β in P2 MEFs transfected with control and *Igfbp5* siRNA. (**D**) Quantitative data for pAkt and pGSK3β levels normalized to level of the corresponding total protein. N=6. (**E**) Representative immunoblots for phospho-Thr202/Tyr204-ERK1/2 (pERK1 and pERK2), ERK1, ERK2, and β-actin in P2, P4, P6, and P8 MEFs. (**F**) Quantitative data for pERK1, pERK2, total ERK1, and total ERK2. N=12 in each passage from five independent experiments. *P<0.05, **P<0.01, ***P<0.001 by one-way repeated measures ANOVA with a Student-Newman-Keuls test. (**G**) Representative immunoblots for pERK1/2, total ERK1/2, and β-actin in P2 MEFs transfected with control or *Igfbp5* siRNA. (**H**) Quantitative data for pERK1, pERK2, total ERK1, and total ERK2 in cells transfected with control or *Igfbp5* siRNA. N=11 in each treatment from six independent experiments. ***P<0.001 by unpaired Student’s t-test. (**I**) Representative immunoblots for phospho-Ser217/221-MEK1/2 (pMEK1/2), total MEK1/2, and β-actin in P2 MEFs transfected with control or *Igfbp5* siRNA. (**J**) Quantitative data for pMEK1/2 and MEK1/2 in cells transfected with control or *Igfbp5* siRNA. N=7 in each treatment from seven independent experiments. *P<0.05 by paired Student’s t-test. Data are represented as mean +/- SEM. kDa: kilodalton. a.u.: arbitrary unit. NS: not significant.

Next, we examined whether downregulation of IGFBP5 modulates intracellular signaling downstream of the IGF1 receptor to induce cellular senescence. Phosphorylation levels of Akt at Ser473 and glycogen synthase kinase 3β (GSK3β) at Ser9, a site phosphorylated by Akt, were increased in P8 MEFs compared with those in P2 MEFs (Figure 4A, 4B). However, knockdown of IGFBP5 resulted in no changes in phospho-Akt (pAkt) and phospho-GSK3β (pGSK3β) levels (Figure 4C, 4D). The levels of pERK1 normalized to total ERK1 were increased in P6 MEFs compared with those in P2 MEFs and were further elevated in P8 MEFs (Figure 4E, 4F). The pERK2 level normalized to total ERK2 was also increased in P6 and P8 MEFs. Knockdown of IGFBP5 in P2 MEFs increased levels of pERK1 and pERK2 (Figure 4G, 4H). IGFBP5 knockdown reduced ERK1 protein level, while ERK2 protein level was unchanged (Figure 4G, 4H). These findings raised the possibility that increased activity of ERK1 and ERK2, but not Akt, is responsible for premature senescence induced by IGFBP5 knockdown. Since the phosphorylation level of MEK1/2, an upstream kinase that phosphorylates ERK1/2, was increased by IGFBP5 knockdown (Figure 4I, 4J), increased activity of MEK1/2 underlies the increases in phosphorylation levels of ERK1 and ERK2 in IGFBP5-knockdown cells.

### ERK2 is involved in senescent phenotypes induced by IGFBP5 knockdown

**Figure 5:**
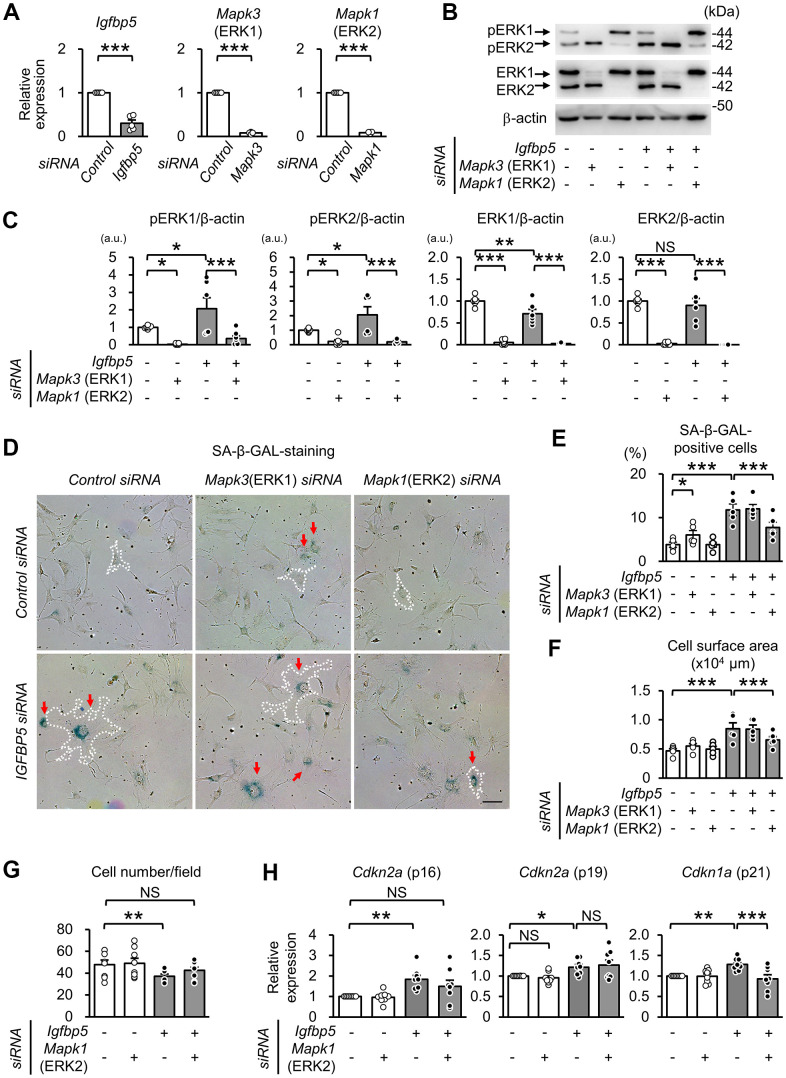
**Effect of knockdown of ERK1 or ERK2 on cellular senescence induced by IGFBP5 knockdown.** (**A**) Levels of *Igfbp5*, *Mapk3* (ERK1), and *Mapk1* (ERK2) mRNA. Knockdown was confirmed by an RT-qPCR method. N=5 in each group. ***P<0.001 by paired Student’s t-test. (**B**) Representative immunoblots for phospho-Thr202/Tyr204-ERK1/2 (pERK1 and pERK2), ERK1, ERK2, and β-actin. P2 MEFs were transfected with control siRNA or *Igfbp5* siRNA followed by additional transfection with either control, *Mapk3* (ERK1), or *Mapk1* (ERK2) siRNA. Cells were analyzed at 48 after transfection. (**C**) Quantitative data for pERK1, pERK2, total ERK1 and total ERK2 levels normalized to β-actin. N=4-7. (**D**) Representative images of SA-β-GAL staining in cells treated as in (**B**). A white dotted line in each field was added to visualize the representative outline of the cell. Red arrows indicate cells positive for SA-β-GAL staining. Scale bar, 100 μm. (**E**) Summary of the percentage of SA-β-GAL-positive cells. N=5 from three independent experiments. (**F**) Summary of cell surface area. N=5. (**G**) Analysis of cell number per field (1449 μm x1087 μm) in MEFs treated as in (**D**). N=8 in each group. (**H**) Levels of *Cdkn2a* (p16 and p19) and *Cdkn1a* (p21) mRNA. N=8 in each group. *P<0.05, **P<0.01, ***P<0.001 by one-way repeated measures ANOVA with a Student-Newman-Keuls test (**C**, **E**–**H**). Data are represented as mean +/- SEM. a.u.: arbitrary unit. NS: not significant.

To examine whether activated ERK1 and ERK2 by IGFBP5 knockdown are involved in the induction of senescent phenotypes, we examined effects of knockdown of ERK1 and ERK2 using a combination with IGFBP5 siRNA in P2 MEFs. Cells were analyzed 48 h after ERK1 or ERK2 siRNA transfection. The RT-qPCR and Western blot analyses confirmed knockdown of ERK1 or ERK2 (Figure 5A–5C). Knockdown of ERK1 alone caused an increase in the level of phospho-ERK2, while knockdown of ERK2 elevated the level of phospho-ERK1 (Supplementary Figure 6). ERK1 knockdown alone increased the number of SA-β-GAL-positive cells. However, increases in SA-β-GAL-positive cells and cell surface area induced by IGFBP5 knockdown were not affected by ERK1 knockdown (Figure 5D, 5E). On the other hand, the increase in SA-β-GAL-positive cells and enlargement of the cell surface area by IGFBP5 knockdown were inhibited by ERK2 knockdown (Figure 5D, 5E). The reduction in the cell number in IGFBP5-knockdown cells was canceled by ERK2 knockdown (Figure 5G). *Cdkn2a* (p16) mRNA level was significantly increased by IGFBP5 knockdown, which was also cancelled by ERK2 knockdown (Figure 5H). ERK2 knockdown did not affect *Cdkn2a* (p19) mRNA level (Figure 5H). *Cdkn1a* (p21) mRNA level in IGFBP5-knockdown cells was reduced by ERK2 knockdown (Figure 5H).

### Roles of IGFBP5 in another type of cellular senescence

We investigated whether downregulation of IGFBP5 is also induced in another type of cellular senescence in MEFs. Since transforming growth factor β (TGFβ) signaling has been shown to be involved in cellular senescence in MEFs [47, 48] and IGFBP5 expression has been reported to be reduced by TGFβ treatment in mouse cells [49], we analyzed P2 MEFs treated with TGFβ. TGFβ treatment increased mRNA levels of *Cdkn2a* (p19) and *Cdkn1a* (p21) and decreased *Igfbp5* expression (Supplementary Figure 7A).

To confirm a change in IGFBP5 expression in human senescence model, we cultured HUVECs and induced replicative senescence by serial passage (Supplementary Figure 7B). The percentage of SA-β-GAL-positive cells was higher at P15 than at P4 (Supplementary Figure 7C). As previously reported [33], IGFBP5 expression level was upregulated in senescent HUVECs (Supplementary Figure 7D).

## DISCUSSION

In the present study, we showed for the first time the role of IGFBP5 in replicative senescence in MEFs. IGFBP5 expression was decreased during serial passage in MEFs (Figure 1F, 1G). Downregulation of IGFBP5 by siRNA in P2 MEFs increased SA-β-GAL-positivity, enlarged cell surface areas, upregulated CDK inhibitors, and suppressed cell proliferation (Figure 2). In contrast, treatment of MEFs with exogenous IGFBP5 delayed the entry to growth arrest (Figure 3A) and reduced the percentage of SA-β-GAL-positive cells (Figure 3B, 3C). These findings suggest that downregulation of IGFBP5 during serial passage contributes to replicative senescence in MEFs. In addition, pERK1 and pERK2 levels were increased in cells transfected with *Igfbp5* siRNA (Figure 4G, 4H) and knockdown of ERK2 attenuated the senescent phenotypes induced by IGFBP5 knockdown (Figure 5D–5H). These results suggest that ERK2 underlies cellular senescence induced by IGFBP5 downregulation.

Our results suggest that downregulation of IGFBP5 during serial passage contributes to replicative senescence in MEFs. Downregulation of IGFBP5 was also observed in the TGFβ-induced senescence model in MEFs (Supplementary Figure 7A). The anti-senescence effect of IGFBP5 in MEFs is opposite to the results of previous studies showing that upregulated IGFBP5 expression contributes to replicative senescence in HUVECs [33] and contributes to premature senescence in human fibroblasts [36]. Upregulation of IGFBP5 in senescent HUVECs was confirmed in the present study (Supplementary Figure 7D). However, as in MEFs in the present study, suppression of proliferation by IGFBP5 knockdown was also observed in human tumor cells [50, 51] and non-tumor cells [52, 53], while the opposite was observed in several lines of cancer cells [54, 55]. Therefore, it seems that the role of IGFBP5 in cellular senescence or cell proliferation may vary depending on the cell type, though additional work is required to clarify this issue.

**Figure 6:**
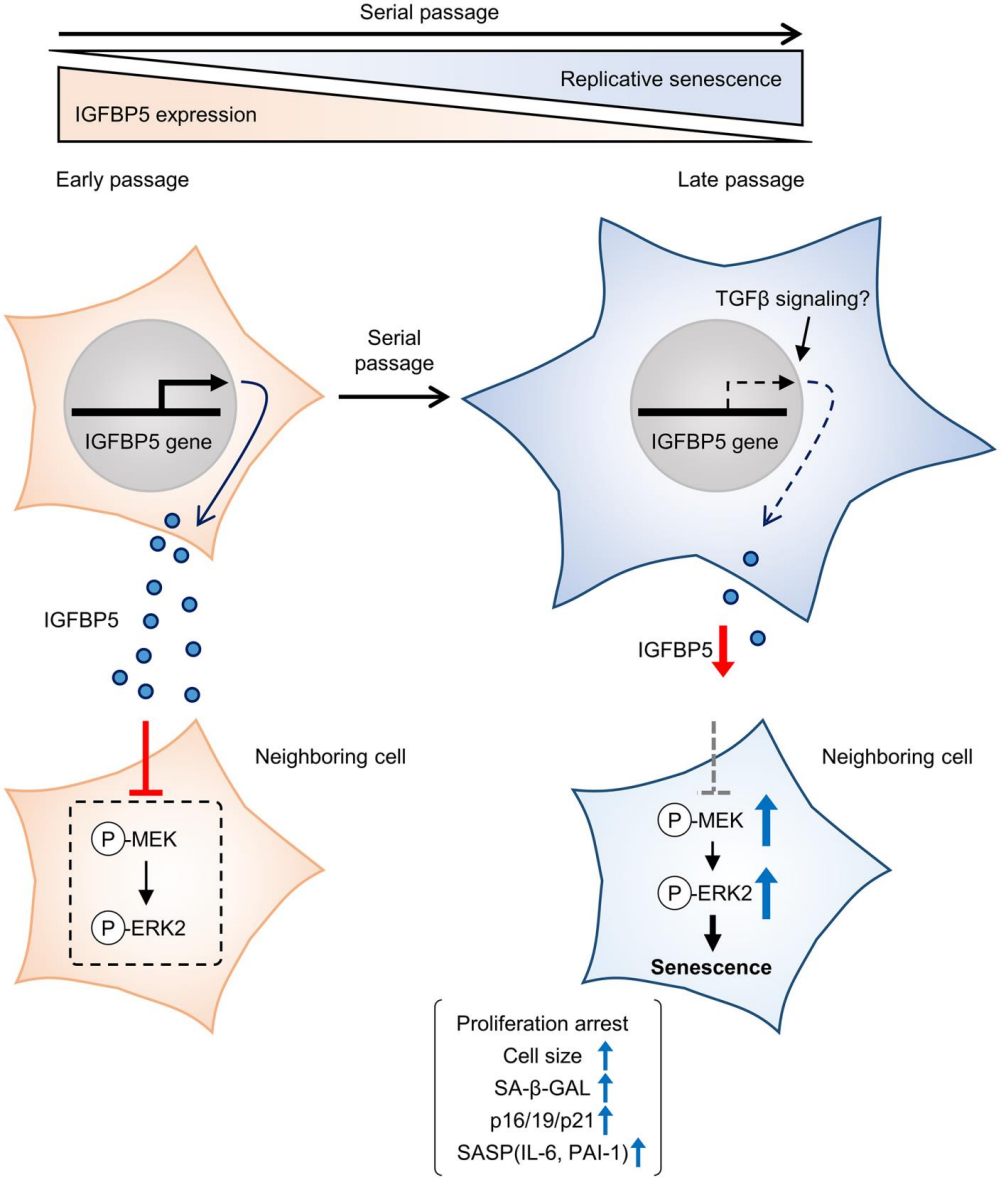
**Schematic summary of our findings.** MEFs at early passage secrete certain levels of IGFBP5. Secreted IGFBP5 proteins inhibit MEK/ERK2 by attenuating their phosphorylation (P) in the neighboring cell, leading to suppression of cellular senescence. IGFBP5 secretion is decreased during serial passage, causing activation of ERK2 and cellular senescence.

IGFBP5 exerts biological effects both extracellularly and intracellularly. Since we found that SA-β-GAL positive cells were similarly detected in cells positive and negative for FAM-labelled siRNA against *Igfbp5* (Supplementary Figure 2), the reduction in intrinsic IGFBP5 level inside the cell does not necessarily promote senescence. On the other hand, exogenous supplementation of IGFBP5 led to the increase in population doubling during serial passage (Figure 3). These findings suggest that cellular senescence during serial passage is attributed to the reduction in levels of extracellular IGFBP5 secreted from the cell rather than intrinsic IGFBP5 inside the cell (Figure 6). On the other hand, we cannot determine whether IGFBP5 released from the cell acts outside the cell or acts after the entry into the cell [21] to inhibit the MEK/ERK pathway and the senescence phenotypes. Further works including immunostaining for IGFBP5 may be required to analyze the relationship between intracellular IGFBP5 level and the senescence phenotype in individual cells.

We found that activated ERK2 mediates induction of cellular senescence in IGFBP5-knockdown cells. Since IGFBP5 knockdown did not change Akt phosphorylation, it is likely that IGFBP5 knockdown preferentially potentiated the Ras/MEK/ERK pathway. Chen et al. showed that IGFBP5 knockdown resulted in increases in ERK1 and ERK2 proteins and their phosphorylation levels [52]. In the present study, protein levels of ERK1 and ERK2 were not increased by IGFBP5 knockdown (Figure 4G, 4H). Instead, IGFBP5 knockdown increased phospho-MEK level (Figure 4I), suggesting that IGFBP5 suppresses ERK1/2 activity via MEK inhibition, though further work is required to clarify how MEK is regulated by IGFBP5.

Our results suggest that ERK2, but not ERK1, is responsible for the increases in the number of SA-β-GAL-positive cells and cell surface area induced by IGFBP5 knockdown. Knockdown of ERK1 alone induced ERK2 phosphorylation (Figure 5B and Supplementary Figure 6) and increased the number of SA-β-GAL-positive cells (Figure 5D, 5E), supporting the notion that activation of ERK2 contributes to the increase in the number of SA-β-GAL-positive cells. Shin et al. showed that ERK2 but not ERK1 mediates Ras-induced proliferation arrest and the increase in cells positive for SA-β-GAL staining in MEFs [16]. Such an ERK2-dominant action has been reported in TGFβ-induced collagen synthesis in NIH/3T3 cells [56]. In the present study, ERK2 was preferentially phosphorylated compared to ERK1 in MEFs (Figures 4E, 4G, 5B), which was commonly observed in MEFs reported by Shin et al. [16] and in NIH/3T3 cells [56]. In addition, Lefloch et al. proposed that the distinct function between ERK1 and ERK2 depends on relative expression levels of ERK1 and ERK2 [57]. Therefore, higher activity of ERK2 than that of ERK1 may explain the dominant role of ERK2 in cellular senescence.

Because IGFBP5 knockdown did not totally mimic senescent MEFs regarding proliferation arrest, SA-β-GAL staining, and upregulation of p16 and p19 (Figure 2), other mechanisms might also contribute to the process of replicative senescence in this model. The expression levels of IGFBP2 and IGFBP7 were upregulated in P8 MEFs (Figure 1F), and both IGFBP2 and IGFBP7 have been reported to promote cellular senescence [30, 32]. Therefore, upregulated IGFBP2 and IGFBP7 might also contribute to replicative senescence in MEFs. On the other hand, downregulation of IGFBP4 in P8 MEFs (Figure 1F) may rather exert an anti-senescence effect because IGFBP4 has been reported to play roles in induction of senescence [32, 58], although the role of IGFBP4 in replicative senescence in MEFs remains unclear.

The mechanism of downregulation of IGFBP5 expression during serial passage is unknown. It has been reported that Ezh2 inhibits IGFBP5 expression [59]. However, Ezh2 expression is rather downregulated in senescent MEFs (Supplementary Figure 5). TGFβ signaling has been implicated in cellular senescence [60] and IGFBP5 expression is reduced by TGFβ treatment in P2 MEFs (Supplementary Figure 7A). Therefore, downregulation of IGFBP5 during serial passage may be mediated via TGFβ signaling.

In conclusion, the results of the present study demonstrated that downregulation of IGFBP5 during serial passage contributes to replicative senescence via an ERK2-dependent mechanism (Figure 6). The results suggest that IGFBP5 counteracts replicative senescence in MEFs.

## MATERIALS AND METHODS

### Cell culture

MEFs were isolated from ddY mice purchased from Sankyo Labo Service Corporation, INC (Tokyo, Japan). Briefly, the head and visceral tissues were removed from embryos and the remaining bodies were finely chopped using a razor blade. The tissues were then incubated twice with trypsin-EDTA at 37° C for 20 min each time. Tissues and cell clumps were dissolved by pipetting, and cells were plated in a 10-cm culture dish (1st passage: P1). In the 3T3 protocol [37], cells were trypsinized and counted every 3 days, followed by replating of 300,000 cells in a 10-cm dish. MEFs were cultured in Dulbecco’s modified Eagle medium containing 10% fetal bovine serum at 37° C in a humidified incubator with 5% CO_2_. Population doubling was calculated by log_2_ (collected cell number three days after seeding) / (seeded cell number). HEK293 cells and COS7 cells were cultured in Dulbecco's Modified Eagle's Medium (4.5 g/L glucose) supplemented with 10% fetal bovine serum and antibiotics. HUVECs were purchased from Cell Applications, Inc. (San Diego, CA, USA). Cells were trypsinized and counted every 5 days and 300,000 cells were plated in a 10-cm dish.

### SA-β-GAL staining and measurement of cell surface area

SA-β-GAL staining was performed using a Senescence Detection Kit (#K320-250, BioVision, Milpitas, CA, USA) according to the manufacturer’s instructions. The percentage of SA-β-GAL-positive cells was determined in each image (500 μm x 500 μm) randomly taken using a KEYENCE/BZ-X700 microscope (Keyence, Osaka, Japan). Data of 15 images from three independent experiments were analyzed in MEFs during serial passage. Cell surface areas were measured by using ImageJ software (https://imagej.nih.gov/ij/).

### RNA analysis

Isolation of total RNA from MEFs and HUVECs and reverse transcriptase reaction were performed as previously reported [61]. Quantitative PCR was performed using GoTaq qPCR Master Mix (A6010, Promega, Madison, WI, USA) and the oligonucleotide primers used are shown in Supplementary Table 1. For analysis of expression of *Il6* in MEFs, the TaqMan gene expression assays (Mm00446190_m1) and TaqMan Universal Master Mix (4369016, Thermo Fisher Scientific) were used. Each sample was run in duplicate and the mean value was used to calculate mRNA expression of the gene of interest and the housekeeping reference gene (18s in MEFs and RPL32 in HUVECs). All assays were performed by the standard curve method using serial dilution of complimentary DNA.

### Western blotting

Western blotting was performed as previously reported [61, 62]. The antibodies used were rabbit polyclonal anti-phospho-Ser473-Akt (#9271, 1:1000, Cell Signaling Technology, Danvers MA, USA), rabbit monoclonal anti-Akt (#4691, 1:1000, Cell Signaling Technology), rabbit polyclonal phospho-Ser9-GSK3β (#9336, 1:1000, Cell Signaling Technology), rabbit monoclonal GSK3β (#9315, 1:1000, Cell Signaling Technology), rabbit polyclonal anti-phospho-Thr202/Tyr204-ERK1/2 (#9101, 1:1000, Cell Signaling Technology), rabbit polyclonal anti-ERK1/2 (#9102, 1:1000, Cell Signaling Technology), rabbit polyclonal anti-phospho-Ser217/221-MEK1/2 (#9121, Cell Signaling Technology), rabbit polyclonal anti-MEK1/2 (#9122, Cell Signaling Technology), rabbit polyclonal anti-p16 (10883-1-AP, 1:500, Proteintech, Rosemont, IL, USA), rat monoclonal anti-p19 (sc-32748, 1:200, Santa Cruz Biotechnology, Santa Cruz, CA, USA), rabbit polyclonal anti-p21 (ab7960, Abcam, Cambridge, UK), goat polyclonal anti-IGFBP5 (sc-6006, 1:200, Santa Cruz Biotechnology), mouse monoclonal anti-FLAG (F3165, 1:1000, Sigma Aldrich; Merck KGaA, Darmstadt, Germany), mouse monoclonal anti-β-actin (010-27841, 1:10000, Fujifilm Wako Pure Chemical Corporation, Osaka, Japan), and mouse monoclonal anti-GAPDH (G8795, 1:10000, Sigma Aldrich, St. Louis, MO, USA).

### Transfection of siRNA

Lipofectamine RNAiMAX Transfection Reagent (13778-150, Thermo Fisher Scientific, Waltham, MA, USA) was used to transfect siRNAs (50 nM) targeting mouse IGFBP5, ERK1, and ERK2 to P2 MEFs according to the manufacturer’s instructions. Mouse *Igfbp5* siRNAs (#1: SASI_Mm01_00183756, #2: SASI_Mm01_00183758, #3: SASI_Mm01_00183760), FAM-labeled siRNA against *Igfbp5* with the same sequence with SASI_Mm01_00183756, mouse ERK1 (*Mapk3*) siRNA (SASI_Mm01_00164575), mouse ERK2 (*Mapk1*) siRNA (SASI_Mm01_00131769) and MISSION^®^ siRNA Universal Negative Control #1 were purchased from Sigma Aldrich. For knockdown of IGFBP5, ERK1 and ERK2, P2 MEFs were first incubated with control siRNA or *Igfbp5* siRNA (#1) for 7.5 h followed by incubation with control, ERK1 or ERK2 siRNA for 7.5 h. Cells were analyzed 48 h after the second transfection.

### Assay for proliferation of cells transfected with IGFBP5 siRNA

MEFs at P2 seeded in a 24-well plate were transfected with control siRNA or siRNA against *Igfbp5*. Cell proliferation was assessed before transfection and at 24, 48, and 72 h after transfection by using Cell Counting Kit-8 (343-07623, Dojindo, Kumamoto, Japan) according to the manufacturer’s instructions.

### Plasmid construction, expression, and purification of IGFBP5

The coding region of mouse IGFBP5 cDNA was cloned by using oligonucleotide primers of 5’-AGGATCCATGGTGATCAGCGTGGTCCTCCT-3’, 5’-TCTCGAGCTCAACGTTACTGCTGTCGAAGG-3’. The PCR fragment was inserted into the BamH1 and Xho1 sites of pIRES-hrGFP-1a Vector (240031, Agilent, Santa Clara, CA, USA). The expression vector for 3xFLAG-tagged IGFBP5 was transfected into cells with Lipofectamine LTX with Plus Reagent (15338100, Thermo Fisher Scientific) according to the manufacturer’s instructions. To isolate and purify FLAG-tagged IGFBP5, the culture medium from HEK293 cells transfected with IGFBP5-FLAG was incubated with anti-FLAG tag antibody beads resin (012-22781, Fujifilm Wako Pure Chemical) at 4° C overnight. The beads were collected by centrifugation and washed three times with 50 mM Tris HCl, pH 7.5, 1 mM EDTA and 150 mM NaCl. IGFBP5-FLAG protein was eluted with 100 mM glycine HCl, pH 3.0 and then neutralized using 1 M Tris HCl, pH 8.0. Protein concentration was assayed by Bradford Reagent (PQ01, Dojindo). Purified IGFBP5-FLAG was confirmed by Western blotting by using an anti-FLAG antibody and was stored in -80° C until use.

### Effects of IGFBP5 on replicative senescence

MEFs cultured with the 3T3 protocol were incubated with purified IGFBP5-FLAG (10 and 30 ng/ml) or a vehicle starting at P2. Treatment of 30 ng/ml IGFBP5 was also started at P4. The concentration of IGFBP5 was determined on the basis of a previous finding that less than 100 ng/ml of IGFBP5 induced a proliferative effect but 200 ng/ml of IGFBP5 had an anti-proliferative action in neuroblastoma cells [63]. Cumulative population doubling was calculated during the 3T3 protocol. At P5, cells were stained with SA-β-GAL and the percentage of SA-β-GAL-positive cells was analyzed. The average percentage was obtained from 12 fields from three independent experiments. Cells at P6 were cultivated for analyses of expression of *Cdk2a* (p16 and p19) and *Cdkn1a* (p21).

### Overexpression of IGFBP5 in MEFs

MEFs at P2 and P4 were transfected with IGFBP5-FLAG by using Lipofectamine LTX with Plus Reagent (Thermo Fisher Scientific) according to the manufacturer’s instructions. Cells were cultivated for analyses 48 h after transfection.

### Analysis of Igfbp5 expression in senescent MEFs in the NCBI database

Changes in *Igfbp5* expression in senescent MEFs were analyzed in accessible Gene Expression Omnibus (http://www.ncbi.nlm.nih.gov/geo/). We searched the GEO repository and found four datasets of DNA microarray which could be analyzed by GEO2R and one RNA sequencing dataset with results normalized into TPM values: GSE39034 (P6-10 vs. <P5) [38]; GSE62369 (P7 vs. P2) [39]; GSE117208 (PD14 vs. PD2) [40]; GSE135258 (P5~6 vs. P3) [41]; and GSE139982 (late passage vs. early passage) [42].

### Treatment of MEFs with a conditioned medium from COS7 cells expressing IGFBP5

COS7 cells cultured in a 10-cm dish were transfected with an empty vector or an expression vector of FLAG-tagged IGFBP5 (15 μg/10-cm dish) using Lipofectamine LTX with Plus Reagent. The conditioned medium and cell lysates were obtained 48 h after transfection. P2 MEFs were incubated with either a control or IGFBP5-transfected conditioned medium for 1 h and were then stimulated with a vehicle or with 10 nM or 20 nM of IGF-1 (791-MG, R & D Systems, Minneapolis, U.S.A) for 10 min. MEFs were cultivated for Western blot analysis of phospho-Akt and Akt levels.

### Induction of senescence by TGFβ in MEFs

P2 MEFs were treated with TGFβ (10 ng/ml, 24 h: 205-16541, Wako Pure Chemicals) to induce premature senescence. Expression levels of *Cdkn2a*, *Cdkn1a*, and *Igfbp5* were analyzed.

### Statistical analysis

Data are presented as means ± SEM. An unpaired or paired 2-tailed Student’s t-test was used to compare two groups. We performed one-way ANOVA or two-way ANOVA and a *post hoc* Student-Neman-Keuls test for multiple comparisons. A difference was statistically significant if p was <0.05. All of the statistical analyses were performed with SigmaPlot (Systat Software, San Jose, CA, USA).

## Supplementary Material

Supplementary Figures

Supplementary Table 1
