# Supplementary material for: Downregulation of IGFBP5 contributes to replicative senescence via ERK2 activation in mouse embryonic fibroblasts
Source: Aging (Albany NY). 2022 Apr 4;14(7):2966–88. doi: 10.18632/aging.203999 (PMC9037271; doi:10.18632/aging.203999)
Supplement: Supplementary Figures [file aging-14-203999-s001.pdf]

SUPPLEMENTARY FIGURES

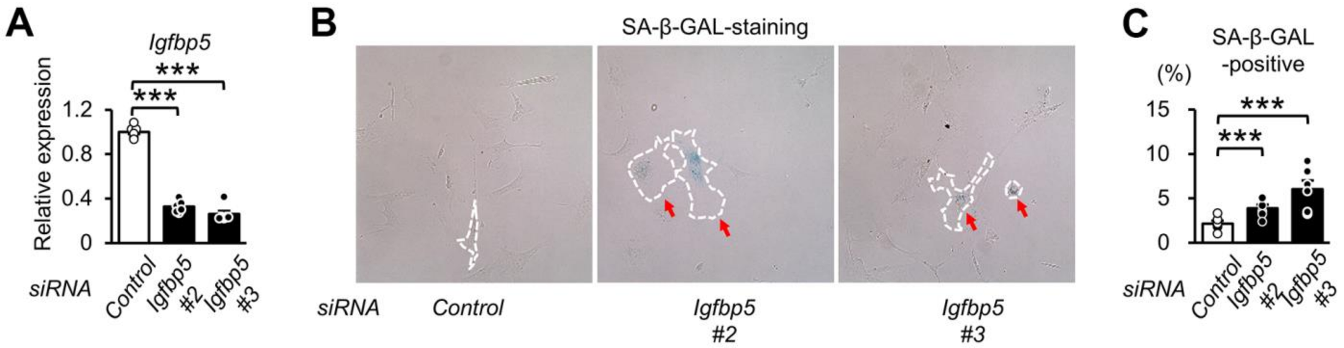

Supplementary Figure 1. Effects of the other sequence of siRNA against Igfbp5 on SA-β-GAL staining. (**A**) Levels of *Igfbp5* mRNA normalize to 18S in P2 MEFs transfected with control siRNA or siRNA against *Igfbp5* (#2 and #3). N=6 in each group. (**B**) Representative images of SA-β-GAL staining 48 h after siRNA transfection. A white dotted line in each field was added to visualize the representative outline of the cell. Red arrows indicate cells positive for SA-β-GAL staining. (**C**) Summary data of the percentage of SA-β-GAL-positive cells. N=6 in each group. ***P<0.001 by one-way repeated measures ANOVA with a Student-Newman-Keuls test for multiple comparisons.

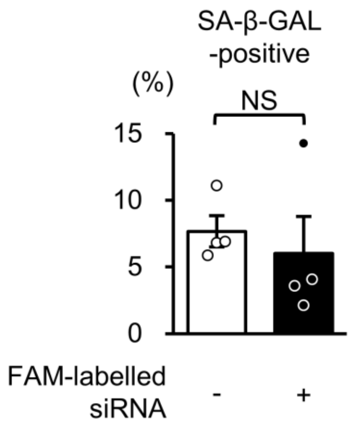

Supplementary Figure 2. Role of intracellular IGFBP5 in cellular senescence. Summary data of the percentage of SA-β-GAL-positive cells in FAM-positive and -negative MEFs. MEFs at P2 were transfected with FAM-labeled siRNA against *Igfbp5.* SA-β-GAL staining was performed 48 h after transfection. N=4 in each group. NS: not significant.

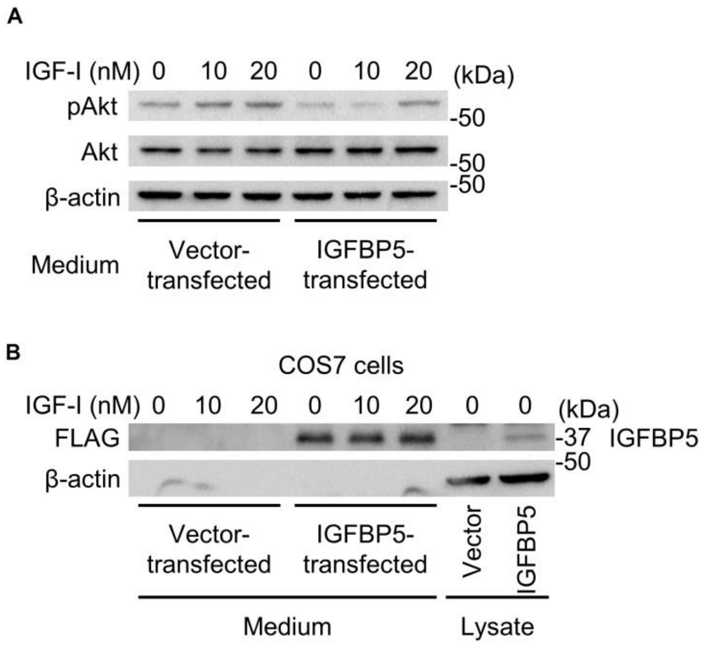

Supplementary Figure 3. Effects of exogenous IGFBP5 on IGF-1-induced Akt phosphorylation. (**A**) Representative immunoblots for phospho-Ser473-Akt (pAkt) and Akt in P2 MEFs treated with indicated concentrations of insulin-like growth factor-1 (IGF-1). Cells were pre-incubated with a conditioned medium from COS7 cells transfected with an empty vector or expression vector of FLAG-tagged IGFBP5. (**B**) Immunoblots for FLAG and β-actin in the medium used in experiments in (**A**) and lysates of COS7 cells transfected with an empty vector or expression vector of IGFBP5-FLAG. kDa: kilodalton.

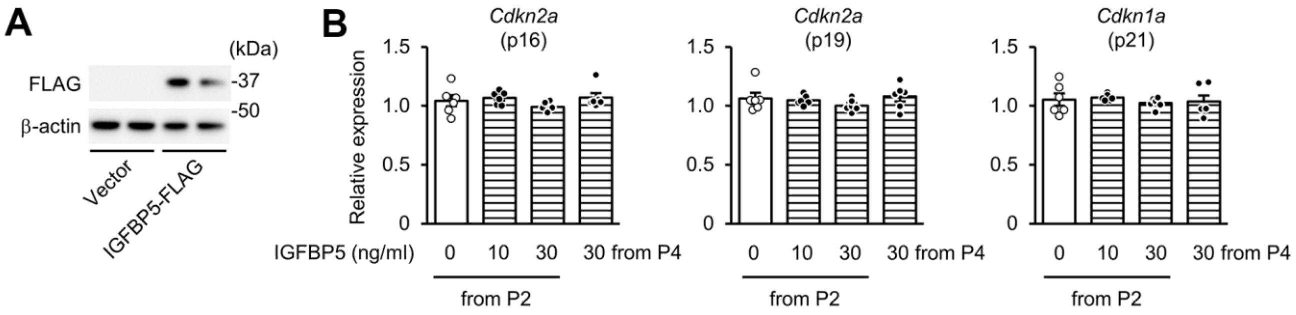

Supplementary Figure 4. Effects of overexpression or exogenous incubation with IGFBP5 on senescence markers. (**A**) Representative immunoblots for FLAG and β-actin of P2 MEFs transfected with an empty vector (Vector) or a vector expressing FLAG-tagged IGFBP5 (IGFBP5-FLAG). (**B**) Levels of *Cdkn2a* (p16 and p19) *and Cdkn1a* (p21) mRNA in P6 MEFs treated with 0, 10, 30 ng/ml IGFBP5 from P2 or 30 ng/ml IGFBP5 from P4. N=6 in each group.

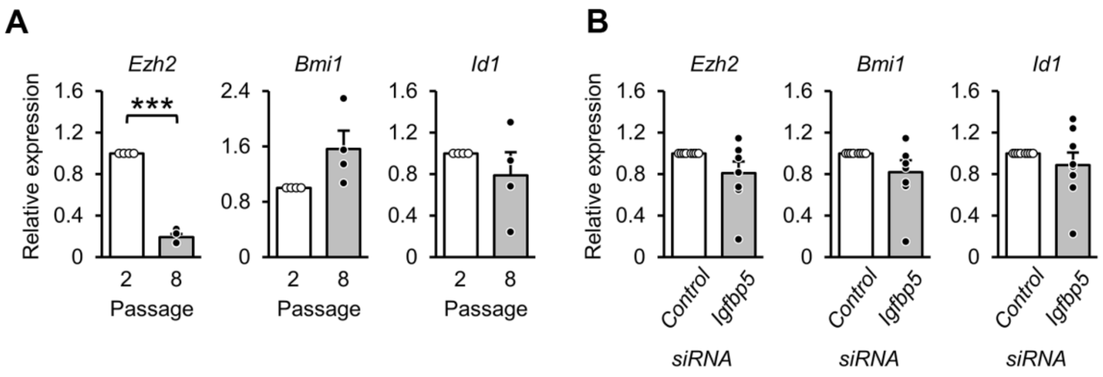

Supplementary Figure 5. Expression levels of p16 repressors. (**A**) Levels of *Ezh2, Bmi1,* and *Id1* mRNA in P2 and P8 MEFs. N=4 in each group. (**B**) Effects of IGFBP5 knockdown on *Ezh2, Bmi1,* and *Id1* mRNA levels. N=8 in each group. ***P<0.001 by paired Student's t-test.

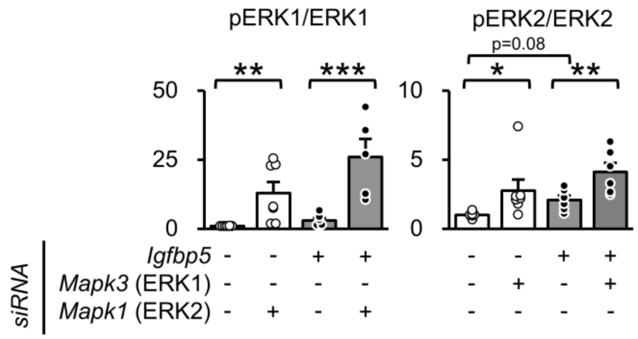

Supplementary Figure 6. Effect of knockdown of *Mapk3* (ERK1) or *Mapk1* (ERK2) on other ERK isoforms. Quantitative data for pERK1 and pERK2 levels normalized to corresponding total protein levels in Figure 5B. N=4-7. *P<0.05, **P<0.01, ***P<0.001 by one-way ANOVA with a Student-Newman-Keuls test for multiple comparisons.

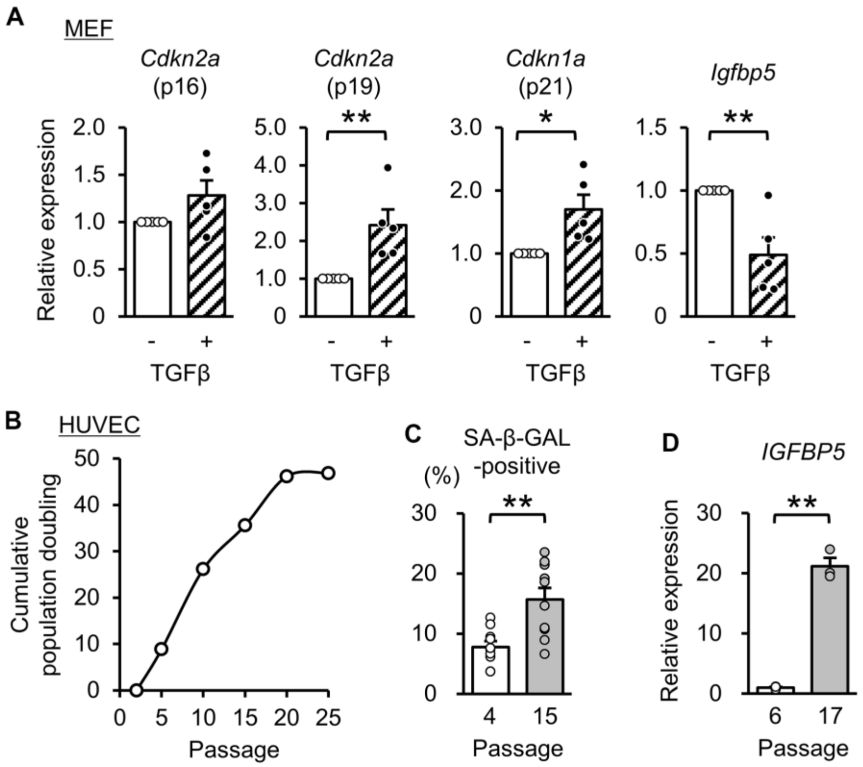

Supplementary Figure 7. Expression level of IGFBP5 in a senescence model induced by TGFβ in MEFs and a replicative senescence model of HUVEC. (**A**) Levels of *Cdkn2a* (P16 and p19), *Cdkn1a* (p21) and *Igfbp5* mRNA in P2 MEFs treated with a vehicle or TGFβ (10 ng/ml, 24 h). N=5 in each group. *P<0.05, **P<0.01 by paired Student's t-test. (**B**) Cumulative population doubling during serial passage in HUVEC. (**C**) Summary data of the percentage of SA-β-GAL-positive cells at P4 and P15. N=10 in each group. (**D**) Levels of *IGFBP5* mRNA in HUVEC at P6 and P17. N=3 in each group. **P<0.01 by unpaired Student's t-test (**C**, **D**).
