# Supplementary material for: Downregulation of IGFBP5 contributes to replicative senescence via ERK2 activation in mouse embryonic fibroblasts
Source: Aging (Albany NY). 2022 Apr 4;14(7):2966–88. doi: 10.18632/aging.203999 (PMC9037271; doi:10.18632/aging.203999)
Supplement: Supplementary Table 1 [file aging-14-203999-s002.pdf]

SUPPLEMENTARY TABLE

Supplementary Table 1. Primer sequences for qRT-PCR.

Gene	Forward	Reverse
**Mouse**		
Cdkn2a (p16)	5'-ATCTGGAGCAGCATGGAGTC-3'	5'-GGGGTACGACCGAAAGAGTT-3'
Cdkn2a (p19)	5'-GCTCTGGCTTTCGTGAACAT-3'	5'-TCGAATCTGCACCGTAGTTGAG-3'
Cdkn1a (p21)	5'-TCCACAGCGATATCCAGACA-3'	5'-GGACATCACCAGGATTGGAC-3'
Igfbp1	5'-CTGCCAAACTGCAACAAGAA-3'	5'-ACACCAGCAGAGTCCAGCTT-3'
Igfbp2	5'-TGGAGGAGTTCCCAGTTTTG-3'	5'-CAGAAGCAAGGGAGGTTCAG-3'
Igfbp3	5'-CAGGCAGCCTAAGCACCTAC-3'	5'-GCATGGAGTGGATGGAACTT-3'
Igfbp4	5'-GACCTGGCTTGGAGTCTGAG-3'	5'-GGCTTATCCTGTAGGGCACA-3'
Igfbp5	5'-CTGCTGGTGTGTGGACAAGT-3'	5'-ACGTTACTGCTGTCGAAGGCGT-3'
Igfbp6	5'-TCCAGTCCACCCAGTTAAGG-3'	5'-CCTTCCAGAGAGTCCAGTGC-3'
Igfbp7	5'-GGAAAATCTGGCCATTCAGA-3'	5'-TGCGTGGCACTCATACTCTC-3'
Serpine1	5'-ACGGTGCTGCCATCAGACTTGTG-3'	5'-ACGCCTGGTGCTGGTGAATGC-3'
Mapk3 (ERK1)	5'-GGCTTTCTGACGGAGTATGTGG-3'	5'-GTTGGAGAGCATCTCAGCCAGA-3'
Mapk1 (ERK2)	5'-TCAAGCCTTCCAACCTCCTGCT-3'	5'-AGCTCTGTACCAACGTGTGGCT-3'
Ezh2	5'-CATACGCTCTTCTGTCGACGATG-3'	5'-ACACTGTGGTCCACAAGGCTTG-3'
Bmi1	5'-ACTACACGCTAATGGACATTGCC-3'	5'-CTCTCCAGCATTCGTCAGTCCA-3'
Id1	5'-TTGGTCTGTCGGAGCAAAGCGT-3'	5'-CGTGAGTAGCAGCCGTTCATGT-3'
18s	5'-CGGACAGGATTGACAGATTG-3'	5'-CAAATCGCTCCACCAACTAA-3'
**Human**		
IGFBP5	5'-ACCTGCTCTACCTGCCAGAA-3'	5'-AGCGAGAGTGCAGGGATAAA-3'
RLP32	5'-CAACATTGGTTATGGAAGCAACA-3'	5'-TGACGTTGTGGACCAGGAACT-3'
